# Genome-Wide Association and Regional Heritability Mapping of Plant Architecture, Lodging and Productivity in *Phaseolus vulgaris*

**DOI:** 10.1534/g3.118.200493

**Published:** 2018-07-02

**Authors:** Rafael T. Resende, Marcos Deon V. de Resende, Camila F. Azevedo, Fabyano Fonseca e Silva, Leonardo C. Melo, Helton S. Pereira, Thiago Lívio P. O. Souza, Paula Arielle M. R. Valdisser, Claudio Brondani, Rosana Pereira Vianello

**Affiliations:** * Department of Forestry; † Department of Statistics, Universidade Federal de Viçosa, Viçosa, MG 36570-000, Brazil; ‡ EMBRAPA Florestas, Colombo, PR 83411-000, Brazil; § Department of Animal Science, Universidade Federal de Viçosa, Viçosa, MG 36570-000, Brazil; ** Common Bean Breeding Program; †† Laboratory of Biotechnology, EMBRAPA Arroz e Feijão, Santo Antônio de Goiás, GO 75375-000, Brazil

**Keywords:** Common beans, RHM QTL, GWAS QTL, DArTseq, Heritability

## Abstract

The availability of high-density molecular markers in common bean has allowed to explore the genetic basis of important complex agronomic traits with increased resolution. Genome-Wide Association Studies (GWAS) and Regional Heritability Mapping (RHM) are two analytical approaches for the detection of genetic variants. We carried out GWAS and RHM for plant architecture, lodging and productivity across two important growing environments in Brazil in a germplasm of 188 common bean varieties using DArTseq genotyping strategies. The coefficient of determination of G × E interaction (c^2^*_int_*) was equal to 17, 21 and 41%, respectively for the traits architecture, lodging, and productivity. Trait heritabilities were estimated at 0.81 (architecture), 0.79 (lodging) and 0.43 (productivity), and total genomic heritability accounted for large proportions (72% to ≈100%) of trait heritability. At the same probability threshold, three marker–trait associations were detected using GWAS, while RHM detected eight QTL encompassing 145 markers along five chromosomes. The proportion of genomic heritability explained by RHM was considerably higher (35.48 to 58.02) than that explained by GWAS (28.39 to 30.37). In general, RHM accounted for larger fractions of the additive genetic variance being captured by markers effects inside the defined regions. Nevertheless, a considerable proportion of the heritability is still missing (∼42% to ∼64%), probably due to LD between markers and genes and/or rare allele variants not sampled. RHM in autogamous species had the potential to identify larger-effect QTL combining allelic variants that could be effectively incorporated into whole-genome prediction models and tracked through breeding generations using marker-assisted selection.

Common bean (*Phaseolus vulgaris*) is a leguminous species reported as one of the most ancient crops in America, with a proposed Mesoamerican origin split into two major centers of genetic differentiation, namely Mesoamerican and Andean gene pools (Bitocchi *et al.* 2012; Schmutz *et al.* 2014). According to CGIAR (Consortium of International Agricultural Research Centers) (CGIAR 2015), approximately 12 million metric tons of common beans are produced annually worldwide, and Latin America is the largest producer, particularly Brazil and Mexico. Common bean is of great social and economic importance, being mostly cultivated by small landholders as well as being an important source of protein, minerals, fiber and carbohydrates (Broughton *et al.* 2003). In Brazil, the common bean is cultivated in three annual harvests, producing approximately 2,670,735 tones on 1,840,696 hectares and estimated productivity of 1,451 kg/ha in 2015. Among the five producer regions, the most prolific are Centre-West (2,036 kg/ha), Southeast (1,621 kg/ha) and South (1,590 kg/ha), accounting for 19%, 29% and 40% of the national production, respectively. The Paraná (1,599 kg/ha) and Goiás (2,438 kg/ha) states are large producers with diverse cropping systems that vary according to edaphoclimatic, socioeconomic and regional market conditions (Souza and Wander 2014).

Agronomic traits such as productivity and plant architecture are determinants in the choice of high-yield bean cultivars. Improved plant architecture and greater tolerance to lodging affect harvest loss, disease occurrence, crop management and mechanized harvest (Teixeira *et al.* 1999). Based on a study conducted over a two-year period by a Nebraska dry bean grower, the total field harvest loss for conventional harvesting was estimated on average at 49,5 kg/ha (Thomas *et al.* 2016). In Brazil, grain loss at harvest time is generally greater than 120 kg/ha, corresponding to ∼8.3% of the total yield (EMBRAPA, 2015). In this case, increased yield at harvest is an important selection criterion for the development of new cultivars (Thung and Rao 1999). Genetic control of plant architecture has been reported to be predominantly due to genes with additive effects (Silva *et al.* 2013), while for yield both additive and non-additive effects have been shown to occur (Nienhuis and Singh 1986). A more detailed knowledge of the molecular basis of these important agronomic traits is critical to improve productivity in common bean.

The application of molecular markers to plant breeding using modern statistical methods (Malosetti *et al.* 2013) has allowed breeders to accurately estimate the positions and effects of genomic regions associated with variation in quantitative traits (Kamfwa *et al.* 2015; Zuiderveen *et al.* 2016; Perseguini *et al.* 2016). This modern approach is very important for bean breeding programs, that are extremely dynamic due to demands of producing regions and consumer markets (Souza *et al.* 2012), and also extremely dependent of genotype-environment interactions (G × E), mainly in terms of tolerance to abiotic and biotic stresses (Beebe *et al.* 2011). Understanding G × E under a molecular level is particularly important for self-pollinating plants that have adapted to constantly changing environments (Li *et al.* 2003), allowing breeders to extrapolate how much of the genetic gain obtained in one environment will be maintained in another one (Eberhart and Russell 1966).

To date, an increasing number of SNP markers has been developed and applied to a diverse set of species at low cost, combining genome complexity reduction with next-generation sequencing (NGS) through RADseq (Willing *et al.* 2011), GbS (Elshire *et al.* 2011) and DArTseq strategies (Cruz *et al.* 2013). For common bean, a few thousand SNPs are currently available, allowing explorations of genetic diversity and population structure (Rodriguez *et al.* 2015; Cichy *et al.* 2015; Valdisser *et al.* 2016). In addition, SNPs have been used in the construction of dense linkage maps (Song *et al.*, 2015), allowing the identification of QTL associated with drought (Mukeshimana *et al.* 2014), agronomic traits (Hoyos-Villegas *et al.* 2016), disease resistance and root architecture (Nakedde *et al.* 2016). GWAS have been successfully used to identify QTL related with biotic (Zuiderveen *et al.* 2016; Perseguini *et al.* 2016), abiotic stress response (Villordo-Pineda *et al.* 2015, agronomic (Kamfwa *et al.* 2015; Moghaddam *et al.* 2016) and technological quality of grains (Cichy *et al.* 2015) have also been identified.

GWAS is already the most popular method to understand the genetic basis of complex traits in plants, since provide new insights on explaining the total genetic variance mainly the presence of small heritability (Thorwarth *et al.* 2017). An analytical approach, called regional genomic relationship mapping or, more simply, RHM, was proposed (Uemoto *et al.* 2013; Nagamine *et al.* 2012) to capture more of the genetic variation potentially associated with traits not detected by GWAS (missing genetic variation). RHM is based on a genomic relationship matrix among individuals based on common and rare SNPs found on small segments of the chromosome, thus combining information from common and rare variants that could be useful to enhance the incorporation of prior knowledge on the underlying genetic architecture. This method has shown high QTL detection power, with low rates of false positives and large fractions of variance explained when compared with other methods by using simulating data (Usai *et al.* 2014). In general, it is expected that RHM regions contain effects large enough to be detected by GWAS that would always be captured by RHM, whereas the opposite would not necessarily be true because of the additional small effect variants accounted for by RHM (Caballero *et al.* 2015; Okeke *et al.* 2018).

To date, studies based on RHM analysis have been conducted with humans (Shirali *et al.* 2015), animals (Riggio *et al.* 2013; Matika *et al.* 2016) and, more recently, with plants (Resende *et al.* 2017). Thus, the aim of this study was to identify and characterize genomic regions that control productivity, lodging and architecture in common bean through GWAS and RHM analyses using SNP markers. In addition, the results of these approaches were compared to determine their performance in capturing additive genetic variation and determining the proportion of trait heritability accounted for by the loci they identified in the common bean genome.

## Material and methods

### Plant material and phenotyping

A total of 188 common bean accessions, including 91 landraces and 97 Brazilian and international cultivars/elite lines belonging to the Andean and Mesoamerican gene pools, were used in the GWAS and RHM for plant architecture, lodging and productivity (S1 Table). However, 580 accessions and four control cultivars were phenotyped in the field. Those 188 accessions effectively used for genotyping and mapping were selected among the initial set of 580 genotypes based on their highest genetic diversity (Valdisser *et al.* 2017). Field experiments were conducted in two important growing environments in Brazil: 1) At Embrapa Arroz & Feijão experimental station (Goiás state), located at 16° 28’ 00” S latitude, 49° 17’ 00” longitude and 823 m altitude; and 2) at Embrapa experimental area in Ponta Grossa (Paraná state) located at 25° 05’ 42” S latitude, 50° 09’ 43” longitude and 969 m altitude.

The experiment was carried out in augmented Federer blocks, in which the plots consisted of three 3-meters rows and four control cultivars with 20 repetitions each. Thus, it was designed 20 blocks with 29 accessions and four controls each. The control cultivars represent a sample of the variability from the main common bean market classes in Brazil. BRS Estilo is a carioca seeded cultivar and BRS Esplendor has black seeds, both with plants of erect architecture (indeterminate growth and type II plants). Jalo Precoce is a jalo seeded cultivar with plants of semi-erect architecture (indeterminate growth and type II plant). BRS Embaixador is from the dark red kidney market class and has plants of erect architecture (determined growth and plant type I). At Embrapa Arroz & Feijão, common bean was cultivated in the winter growing season from April to June (2010) with average rainfall of 51.8 mm, average relative humidity around 60–70% and average temperature of 21.8°, ranging from 16° to 29° (Silva *et al.* 2010). At the experimental area in Ponta Grossa, common bean was cultivated in the dry growing season from December to April (2011) with average rainfall of 143.6 mm, average relative humidity around 50–60% and average temperature of 19.96°, ranging from 17.2° to 21.4° [https://pt.climate-data.org/location/4493/].

The winter season at Embrapa Rice and Beans Goiás (GO) and the dry season in Ponta Grossa (PR) represent distinct conditions for common bean cultivation. Winter growing season is carried out using high technology, including irrigation by central pivot, chemical fertilization and chemical control of pests. Soil diseases are more likely during winter, thus there is no relevant abiotic stress on this growing season, consequently leading to higher productivity in this period. In the dry season at experimental area in Ponta Grossa, common bean is cultivated without supplementary irrigation. Although the main diseases during the dry season are anthracnose (*Colletotrichum lindemuthianum*), angular leaf spot (*Pseudocercospora griseola*) and some bacterioses, the drought is the major abiotic stress that affects the yield in this condition.

Agronomic trait architecture was determined by measuring branch insertion angles, length of guide and stem height at which all pods are above the soil surface; lodging tolerance was determined by the degree of inclination in relation to vertical. Both traits were observed at harvest maturity and evaluated on a scale from 1 (short guides, high pods, closer branches and without lodging) to 9 (long guides, low pods, open branches and lodged plants) as previously described by others (Melo *et al.* 2009). Productivity was determined by the weight of the beans harvested from each plot, and grain yield was expressed in kg ha^-1^ at 13% humidity. Phenotypic correlations between the pairs of traits were given by the *Pearson* correlation at a confidence interval of 95%

### Phenotypic modeling and genetic parameters

The adjustment of the phenotypic data were performed using the free software SELEGEN-REML/BLUP (Resende 2007), employing the augmented block model described in equation 1 below:

y=Xf+Zg+Sb+Tk+ε, [1]

where y is the data vector, f is the vector of the assumed fixed effects (means of the reference genotypes and mean of the population of main treatments at each site), g is the vector of the genotypic effects of the varieties (assumed to be random), b is the vector of the environmental effects of the blocks (assumed to be random), k is the vector of the effects of the genotype × environment interaction (random), and ε is the vector of the residual effects (random). The capital letters **X**, Z, S and T represent the incidence matrices for these effects. The vector of the genetic effects g can be divided into two groups: varieties and checks. The first group is given as random, with a multivariate normal distribution, a mean of zero and a covariance matrix $A\sigma_{g}^{2}$, where A is the additive genetic kinship matrix and $\sigma_{g}^{2}$ is the genetic variance among the varieties of *P. vulgaris*. The second group is not relevant to the detection of QTL because its individuals are included in the model as a fixed effect to help correct for non-genetic effects (Pastina *et al.* 2012). The corrected phenotypes used in the QTL mapping methods (GWAS and RHM) are given by $y^{*}=\hat{g}/r_{g\hat{g}}{}^{2}$, where $r_{g\hat{g}}$ is the $\hat{g}$.

In order to contemplate the relevance of the G × E interaction, the respective variance component was included in the numerator of the joint heritability (${\text{h}}_{\text{joint}}^{2}$) aiming to evaluate the genetic behavior of the varieties over the two experimental conditions. This mentioned heritability was calculated as follows: $h_{joint}^{2}=(\sigma_{g}^{2}+\sigma_{k}^{2})/(\sigma_{g}^{2}+\sigma_{k}^{2}+\sigma_{b}^{2}+\sigma_{\varepsilon }^{2})$, where $\sigma_{g}^{2}$, $\sigma_{k}^{2},\;\sigma_{b}^{2}$ and $\sigma_{\varepsilon }^{2}$ are the components of variance between the varieties, the components of the interaction variety × environment, and the between blocks and residual effects, respectively. The coefficient of determination of the G × E interaction is given by $c_{int}^{2}=\sigma_{k}^{2}/(\sigma_{g}^{2}+\sigma_{k}^{2}+\sigma_{b}^{2}+\sigma_{\varepsilon }^{2})$, and the genetic correlation coefficient between sites is defined as $r_{\mathrm{gl}}=\sigma_{g_{1},g_{2}}/\sqrt{\left(\sigma_{g_{1}}^{2}+\sigma_{g_{2}}^{2}\right)}$, where $\sigma_{g_{1},g_{2}}$ is the genetic covariance between the two sites, and $\sigma_{g_{1}}^{2}$ and $\sigma_{g_{2}}^{2}$ are the genetic variances at each of the two sites.

### Genotyping and analysis With DArTseq method

In addition to analysis of SNPs generated by DArTseq method (Sánchez-Sevilla *et al.* 2015) we also utilized SilicoDArT (DArT) markers which were extracted from the DArTseq libraries by DArTsoft14 pipeline. While a proportion of SilicoDArT markers is based on SNP polymorphism in restriction enzyme (RE) recognition sequences and therefore simply add to SNPs extracted from the sequenced tags, there were also additional sources of SilicoDArT polymorphisms, namely InDels in recognition sites and within the tags (restriction fragments) and, also, methylation variation at the genome level. The genome complexity reduction was achieved by using the RE *PstI* and Mse*I*. The DArT were genotyped as Presence Absence Variations (PAVs). DArT and SNP marker qualities were determined by reproducibility and call rate scores.

### Linkage disequilibrium (LD)

The squared correlation coefficient (*r*^2^) was used as LD measure over all markers pairs. The LD concept was relevant to inform an appropriate window length for RHM. When omitting the LD analysis, the windows length to be used in RHM can be empirically assumed, which decrease the power to detect true QTL regions. Additionally to *r*^2^, the LD estimates were corrected for bias due to population structure and relatedness ($r_{s}^{2}$) using the LDcorSV (Mangin *et al.* 2012). Genetic structure was inferred by Structure v 2.3.4 (Pritchard *et al.* 2000) and the most likely K was determined (∆K) as previously proposed (Evanno *et al.* 2005) using the program Structure Harvester (Earl 2012). LD decay curves were adjusted using a standard exponential function.

### GWAS Analysis

The following model was used in the GWAS:

$$y^{*}=\text{X}\beta +Zu+m_{i}+\varepsilon ,$$ [2]

where $y^{*}$ is the vector of phenotypes adjusted using the model described by equation 1; β is the fixed effect of intercept or general mean; u is the additive random effect of genotype/variety; $m_{i}$ refers to the fixed effect of the **i**^th^ marker; and ε is the residual effects of the model. The distributions and covariance structures of u and ε are given by $\text{u}|{\text{σ}}_{\text{u}}^{2}∼\text{N}\left(0,G{\text{σ}}_{u}^{2}\right)$ and $\varepsilon |{\text{σ}}_{\varepsilon }^{2}∼\text{N}(0,I{\text{σ}}_{\varepsilon }^{2})$, respectively, where I is an identity matrix and G is the complete genomic kinship matrix, which can be represented based on the following equation [3]:

$$G=\frac{{WW}^{T}}{\sum_{1}^{m_{i}}2p_{i}(1-p_{i})},$$ [3]

where W is the incidence matrix formed by the SNP and DArT markers (sorted based on their position in the genome), assuming $W_{\text{SNP}}\subset \{-1,0,1\}$ and $W_{\text{DArT}}\subset \{-1,1\}$, $p_{i}$ is the allele frequency of the **i**^th^ marker in the matrix  W. The determination of the significance of the loci markers was performed as described in the RHM method.

### RHM Analysis

QTL mapping using the RHM method was performed as previously described in the literature (Nagamine *et al.* 2012; Resende *et al.* 2017). In summary, RHM provides heritability estimates for genomic segments containing both common and rare allelic effects. Additionally, this model assumes G × E interaction, that enhance the knowledge on regional genetic variation related to environment adaptation. This mentioned RHM model presented in equation (4) was fitted using the *regress* (Clifford and McCullagh 2012) R package:

$$y^{*}=\text{X}\beta +Z_{1}u+Z_{2}v+\varepsilon ,$$ [4]

where $y^{*}$, u, ε, β and X are the same as described for the GWAS method, and v is the random regional genomic additive effect. The distribution and covariance structure of v is given by $\text{v}|{\text{σ}}_{v}^{2}∼\text{N}\left(0,G_{REG}{\text{σ}}_{v}^{2}\right)$. $G_{REG}$ is a matrix similar to G (equation 3), but using a subset of the matrix W. These subsets were determined by genomic “windows” or “regions” of two Mb in length overlapping by one Mb (*e.g.*, the three first regions are 0-1, 1-2 and 2-3 Mb) matching the estimated LD (see results). To test for the presence of regional variance (${\text{σ}}_{v}^{2}$) against the null hypothesis of no regional variance, a likelihood ratio test (LRT) was used, where L0 and L1 represent the likelihood values for the hypothesis of absence (H0) or presence (Ha) of regional variance respectively (*i.e.*, the complete model of equation 4 *vs.* the reduced model without ${\text{σ}}_{v}^{2}$).

To explore the statistical test distribution of the null hypothesis and find the optimal threshold for each trait, the same permutation test with Bonferroni correction for multiple tests with a global α=0.05 used for GWAS was used for RHM. Genomic segments displaying significant ${\text{σ}}_{v}^{2}$ were declared as regional QTL. The same whole-genome relationship matrix G was used to analyze all regions, differently from ${\text{G}}_{\text{REG}}$ that varies over the genome positions. The G matrix used here (VanRaden 2008) is an efficient method for processing genomic data by increasing the reliability of estimated breeding values in the presence of thousands of marker effects simultaneously. Because any consequential correlation generated between whole-genome and regional relationships would be very small and would tend to reduce the likelihood of detecting regional effects (Nagamine *et al.* 2012), the likelihood of a detected regional effect should increase when the same matrix is used for all regions. Otherwise, fitting the whole-genome relationship matrix G with a random effect accounts for relatedness and family structure.

Whole genome heritability is given by $h_{G}^{2}=\sigma_{u}^{2}/(\sigma_{u}^{2}+\sigma_{\varepsilon }^{2})$, using the variance components of the reduced model of equation (4), *i.e.*, the model fitted without the regional genomic window components v. The regional heritabilities to each genomic window segment are $h_{RHM}^{2}=\sigma_{v}^{2}/(\sigma_{v}^{2}+\sigma_{u}^{2}+\sigma_{\varepsilon }^{2})$, in the full model of equation [4]. These estimates were separately calculated for the DArT, SNP and the combined set of markers (SNP+DArT).”

### Effects of allelic substitution

The average effects of allelic substitution at the identified QTL with no environmental interaction were statistically analyzed for overall significance using Welch’s *t*-test as implemented by R software version 3.1.3 (Welch 1947).

### Identification of transcripts in regions containing joint-trait QTL

The QTL identified through GWAS and RHM joint analyses were placed into haplotype blocks previously identified for common bean (Valdisser *et al.* 2017), and nearby genes were identified using the common bean genome annotation available at Phytozome.

### Data availability

The genome sequence and annotations are available at [https://phytozome.jgi.doe.gov/pz/portal.html]. Figure S1 presents the G matrices representing genomic relationship coefficients of *P. vulgaris* based on SNP and DArT markers. Figure S2 shows detailed genomic information of the association of DArT marker with lodging trait. Table S1 contain the list of common bean genotypes used in the molecular and phenotypic characterization. Table S2 presents the description of the functional annotation of the significant QTL detected in the joint analysis by RHM. Table S3 describe the summary of total trait and genomic heritability as well as the fractions captured by the markers in the analysis. Files containing the phenotypic and genotypic data used to perform the association analysis are available at Figshare. Supplemental material available at Figshare: https://doi.org/10.25387/g3.6533933.

## Results

### Phenotypic evaluation of the experiment

Information on the fit of model 1 as well as the genetic parameters (genetic variance components, joint and regional heritabilies) of the two experiments is shown in Table 1. At site 1 (Goiás) none of the plots were lost; and all varieties (192 in total, given by 188 + 4 reference genotypes) provided phenotypic values for the analysis. In site 2 (Paraná), phenotypic information for architecture and lodging was collected for 136 varieties (132 + 4 reference genotypes); whereas for architecture a total of 190 (186 + 4) varieties were evaluated. For the traits plant architecture and lodging tolerance the obtained overall average values were 4.61 with standard deviation (SD) of 1.097 and 4.44 with SD of 1.634, respectively. Variations were observed in the means due to the environments, being more favorable for the environment of Goiás in the Central-West region (4.36 architecture and 4.32 lodging), compared to Paraná in the South region (4.96 architecture and 4.60 lodging), which was anticipated considering their geographic differences and the different crop yields. For productivity, the average value obtained for the combined experiments was 1,342 ± 653. A set of 70 and 66 accessions had above average values in Goiás (1,544 kg/ha) and Paraná (1,585 kg/ha), respectively, including 27 accessions (8 elite lines/cultivar and 19 traditional varieties) common to both environments with averages of 1,956 kg/ha (Goiás) and 2,080 kg/ha (Paraná). Correlation coefficients between plant architecture traits and productivity were weak and negative (*P* ≤ 0.05), ranging from -0.14 between productivity and lodging, to -0.18 between productivity and architecture, whereas between architecture and lodging it was a positive significant correlation (0.75).

**Table 1:** Genetic parameters (with standard deviation - SD) and phenotypic adjustment for the traits plant architecture (arch.), lodging (lodg.) and grain productivity (prod.) for common bean, estimated separately for each environment and in joint analysis, considering the 188 genotypes used in the GWAS and RHM

Local	Parameter	Arch.(SD)	Lodg. (SD)	Prod. (kg/ha) (SD)
Goiás	**Num. of inbred lines**	192	192	192
	**Phenotypic mean**	4.36 (0.09)	4.32 (0.08)	1544.60 (140)
	**h^2^ plot means**	0.82 (0.10)	0.77 (0.07)	0.30 (0.04)
	$r_{g\hat{g}}$*	0.91 (0.08)	0.88 (0.06)	0.55 (0.03)
Paraná	**Num. of inbred lines**	136	136	190
	**Phenotypic mean**	4.96 (0.10)	4.60 (0.11)	1585.42 (145)
	**h^2^ plot means**	0.82 (0.12)	0.87 (0.09)	0.57 (0.05)
	$r_{g\hat{g}}$	0.91 (0.09)	0.93 (0.08)	0.75 (0.08)
Combined	**h^2^ plot means**	0.72 (0.05)	0.67 (0.04)	0.03 (0.001)
	$r_{g\hat{g}}$	0.85 (0.08)	0.82 (0.06)	0.17 (0.02)
	$h_{\mathrm{total}}^{2}$	0.81 (0.05)	0.79 (0.04)	0.43 (0.05)
	$c_{\mathrm{int}}^{2}$	0.17 (21%)	0.21 (27%)	0.41 (95%)
	$r_{\mathrm{gl}}$	0.77 (0.10)	0.73 (0.11)	0.04 (0.002)
	**Num. of Genotyped lines**	181	181	181
	***h*^2^_G_ SNP**	0.76 (0.09)	0.75 (0.09)	0.32 (0.04)
	**Explained herit. (%)**	93.83%	94.94%	74.42%
	***h*^2^_G_ DArT**	0.80 (0.11)	0.76 (0.08)	0.28 (0.06)
	**Explained herit. (%)**	98.77%	96.20%	65.12%
	***h*^2^_G_ SNP+DArT**	0.81 (0.08)	0.79 (0.07)	0.31 (0.03)
	**Explained herit (%)**	≈100.00%	≈100.00%	72.09%

$r_{\text{g}\hat{\text{g}}}:$ selection accuracy; *h*^2^*_total_*: joint heritability; c^2^*_int_*: coefficient of determination of G × E interaction; $r_{\mathrm{gl}}$: genetic correlations within a trait across environments; *h*^2^ plot means: trait heritability of the germplasm data; *h*^2^_G_: whole genome heritability based on molecular data.

Between the two experiments, estimates of accuracy in genotype selection ($r_{g\hat{g}}$ ≥ 88%) showed high experimental quality and thus confidence in the ability to select for superior genotypes for architecture and lodging. For the trait productivity, accuracy estimates were reduced for the environment in Goiás (55%) and for the joint analysis (17%). The heritabilities, both at the mean level (*h*^2^ plot means**)** and at the level of the individual plots, were more similar between the environments for plant architecture (0.82), whereas for the traits lodging (0.87) and productivity (0.57), the highest estimates were obtained for the cultivation environment in the south of the country (Paraná). The differences between the heritabilities at the individual and average plot levels were very similar for all traits.

The parameter $c_{int}^{2}$ represents how much total heritability ($h_{\text{total}}^{2}=h_{\text{g}}^{2}+{\text{c}}_{\text{int}}^{2}$) is due to the genetic interaction between sites. In this sense, it is possible to observe that the traits plant architecture and lodging had low values for $c_{int}^{2}$ compared to the trait productivity, indicating that the environment had a much more significant impact on productivity than on the other two traits. In addition, the values for $r_{gl}$ indicate that the genotypes behaved very similarly at both sites with respect to the traits plant architecture and lodging. In contrast, the most productive genotypes at Goiás were not the same as those at Paraná ($r_{gl}=0.04$).

### Molecular marker genotyping and LD

For DArT, a total of 11,564 polymorphic markers were genotyped. These markers presented an average call rate of 95.2%, indicating less than 5% missing data. The average reproducibility score was 99.9%, with a result of 100% for the vast majority of markers (95.6%) and the lowest value estimated at 96.8%. A total of 6,286 SNPs from DArTseq were identified with an average call rate of 92% (∼8% missing data) and an average reproducibility score of 99.43%. Of the 17,850 markers evaluated, 6677 (3443 DArT and 3234 SNP) were identified in linkage equilibrium (37.4%). Estimated heterozygosity was lower than 1% in almost all chromosomes, with an average value of 0.89% (Table 2), indicating the similarity of informativeness between dominant and codominant markers in autogamous plants.

**Table 2:** Distribution, heterozygosity and linkage disequilibrium (LD, *r*^2^) of molecular markers (SNP and DArT), genotyped and analyzed individually for all chromosomes

Chromosome	Length (Mb)	Marker count			SNP Heterozygosity	Average LD (*r*^2^)		
		SNP	DArT	Total		SNP	DArT	SNP+DArT
1	52.02	320	286	606	0.98%	0.0378	0.0364	0.0361
2	49.01	458	409	867	0.76%	0.0594	0.0610	0.0594
3	52.18	358	364	722	0.90%	0.0441	0.0371	0.0399
4	45.80	179	260	439	0.86%	0.0615	0.0432	0.0494
5	40.57	251	282	533	0.84%	0.0379	0.0320	0.0338
6	31.95	287	250	537	1.01%	0.0445	0.0430	0.0432
7	51.71	307	299	606	0.83%	0.0405	0.0351	0.0367
8	59.63	318	391	709	0.96%	0.0367	0.0314	0.0331
9	37.42	315	295	610	0.87%	0.0302	0.0276	0.0280
10	43.25	197	261	458	1.03%	0.0321	0.0345	0.0326
11	50.00	244	346	590	0.80%	0.0699	0.0463	0.0539
**Total**	**513.54**	**3234**	**3443**	**6677**	**0.89%**	**0.0360**	**0.0330**	**0.0312**

**Figure 1:**
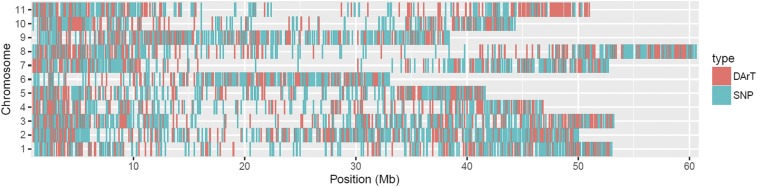
Distribution of 3443 DArT and 3234 SNP markers (6677 total markers) along the 11 chromosomes of common bean (y-axis). The x-axis represents chromosome position in Mb.

The molecular markers were aligned with reference to the 11 chromosomes of the reference genome of *P. vulgaris* as represented in Figure 1. Both classes of markers presented a genome-wide distribution. The average number of SNPs per chromosome was 607, ranging from 439 on chromosome 4 to 867 on chromosome 2 (Table 2, Figure 1). A high density of markers was observed at the ends of each chromosome except for chromosome six, which showed a lower density at the initial portion. Chromosomes 1, 3, 7, 10 and 11 showed reduced marker density in the centromere region.

**Figure 2:**
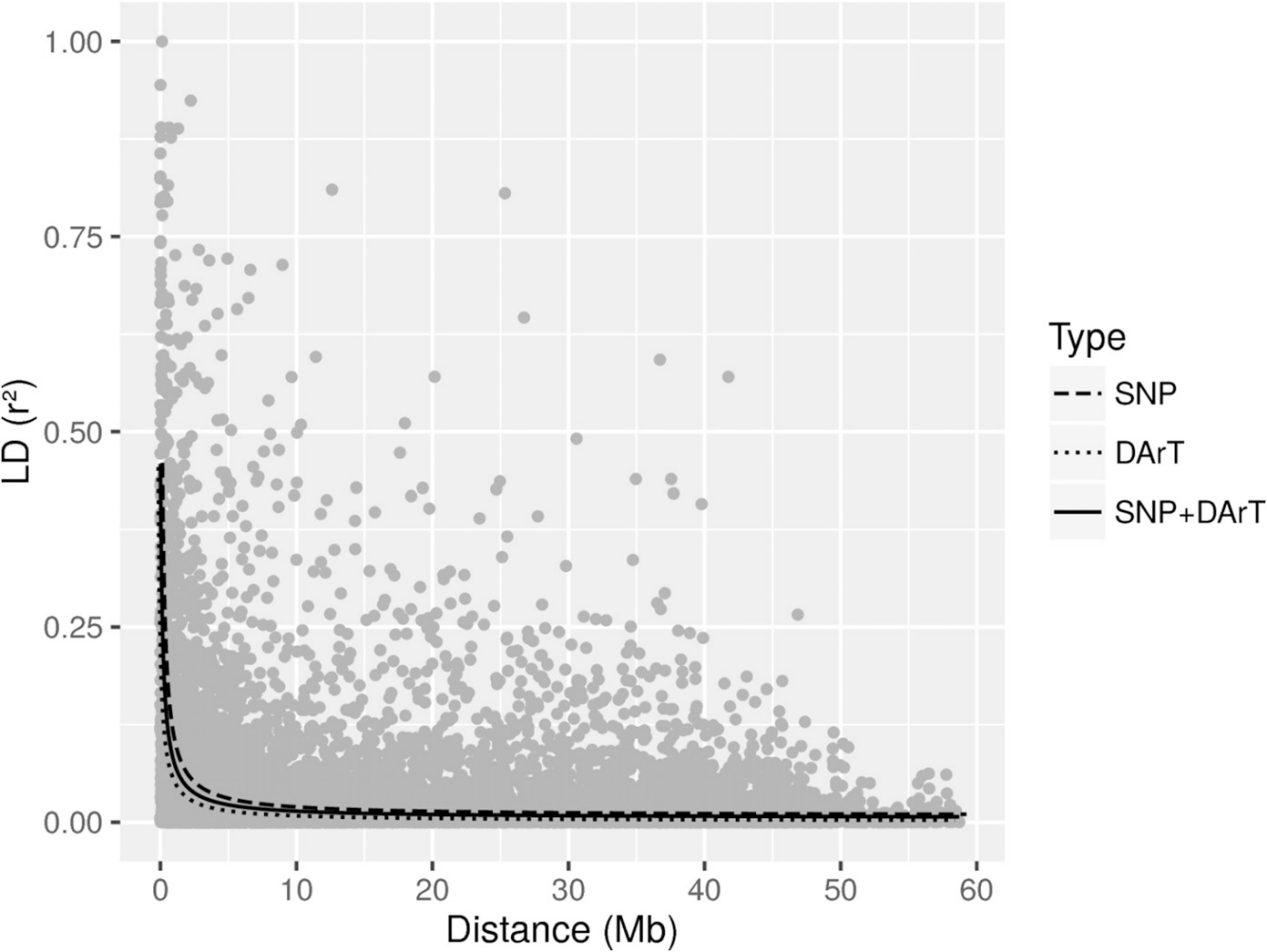
Allele pair linkage disequilibrium (*r*^2^) across the entire *P. vulgaris* genome for all genotypes, plotted according to genetic distance in Mb and including both DArT and SNP markers. Decay lines for DArT (dotted line), SNPs (dashed line) and both marker types (solid line) are shown.

The relatedness matrix including all markers revealed two distinct groups representative of the Andean and Mesoamerican gene pools (S1 Fig), represented by 69 and 112 accessions, respectively. The average *r*^2^ for the full sample set and all markers (6677) corrected for relatedness and structure showed a faster rate of decay and dropped to half at a pairwise *r*^2^ value of 0.0312, estimated at 0.033 for DArT and 0.036 for SNP (Figure 2). An abrupt drop in LD decay was observed in the intervals spanning 1 to 2 Mb for the markers analyzed separately or together, which therefore seemed to be an appropriate window length for RHM.

### Detection of associations via GWAS

The significance of genome association subjected to multiple testing corrections are described in Table 3 and provide an overview of the positioning of the markers, the fractions of the genomic heritabilities and the threshold [-*log10* (*p*-value)] of the identified QTL. A total of 60,093 association tests were performed (6,677 markers × 3 traits × 3 sites, *i.e.*, site 1, 2 and the combination of both sites). After the multiple testing corrections, eight QTL were identified for the three traits evaluated that were associated with three markers. For the traits architecture and lodging, the two QTL identified in Goiás and Paraná, as well as in the joint analysis, were associated with the marker DArT X3366188 located on chromosome 1 (position at 15.72 Mb). The significance of the identified QTL [−*log10* (*p*-value)] ranged from 3.95 (environment 1) to 4.53 (joint analysis). Fractions of explained genomic heritabilities were high, capturing up to 28.4% of additive genetic variation for architecture and 30.4% for lodging. For the trait productivity, two QTL were identified in environment 1. The QTL were associated with two closely positioned SNP markers on chromosome 3 (X3382215 and X3382284), with significant associations [–log_10_(p)] above 4.06 and fractions of explained heritabilities above 20%. All fixed QTL effects (m_i_) were positive.

**Table 3:** Molecular markers and associated trait loci in common bean germplasm according to genome-wide association study (GWAS) for three traits, analyzed by environment and jointly

Local	Trait	Chrom.	Position	Type	Marker Code	2pq	*h*^2^_GWAS_	Total *h*^2^ explained^£^	–log_10_(p)
Joint	Architecture	1	15.72	DArT	X3366188.F.0	0.46	0.23	28.4%	4.31
	Lodging	1	15.72	DArT	X3366188.F.0	0.46	0.24	30.4%	4.53
Goiás	Architecture	1	15.72	DArT	X3366188.F.0	0.46	0.19	23.2%	4.16
	Lodging	1	15.72	DArT	X3366188.F.0	0.46	0.23	30.0%	4.33
	Productivity	3	48.15	SNP	X3382215.F.0	0.47	0.06	20.0%	4.06
	Productivity	3	49.18	SNP	X3382284.F.0	0.48	0.07	23.3%	4.89
Paraná	Architecture	1	15.72	DArT	X3366188.F.0	0.46	0.19	23.2%	3.98
	Lodging	1	15.72	DArT	X3366188.F.0	0.46	0.18	20.7%	3.95

2pq: frequency of the heterozygous genotype of the associated marker; £: percentage of *h*^2^ trait explained by GWAS QTL (*h*^2^_GWAS_/*h*^2^_G_); –log_10_(p): *p*-value expressed as -log10; Chrom: chromosome.

### Detection of associations via RHM

A total of 478 genomic windows were subjected to RHM analysis, each one covering a variable number of polymorphic SNPs and DArTs providing a coverage density of approximately one marker every 76.9 kb. Across the genome, regions spanned a minimum of two and a maximum of 107 markers. The mean and median number of SNPs within a region were 27.2 and 22, respectively.

A set of 13 regional QTL were mapped considering a total number of markers varying from 3 to 41 across 5 chromosomes (Table 4). Six QTL were identified in chromosomes 1, 8, 9 and 11 as controlling plant architecture. QTL at chromosomes 1 (spanning 8 SNPs) and 8 (spanning 41 SNPs) were found in the Goiás environment and in the joint analysis. Explained RHM heritability ranged from 22 to 33%. For the plant lodging, three RHM QTL were identified in both environments through separated and joint analysis. The explained heritability ranged from 34.5 to 49.4%, with a minimum [-log10 (p)] of 2.69 in Paraná. This RHM QTL overlapped with an architecture-related QTL identified in Goiás environment and in the joint analysis. For plant productivity, RHM QTL were detected in chromosomes 1, 4 and 8 in both environments and the joint analysis, spanning from three (chrom. 8) to 31 SNPs (chrom.1), explaining from 11.6 to 40% of the genetic variance with minimum and maximum significances of 2.89 and 3.79, respectively.

**Table 4:** Identification of QTL intervals in common bean germplasm through regional heritability mapping (RHM) using 2 Mb genomic segments with 1-Mb sliding window and their contribution to the additive genetic variance for traits plant architecture (arch.), lodging (lodg.) and grain productivity (prod.) evaluated by environment and jointly

Local	Trait	Chrom.	Region start position (Mb)^€^	Region end position (Mb)^€^	Num. of Markers	*h*^2^_RHM_	Total *h*^2^ explained^£^	–log_10_(p)
Joint	Architecture	1	15.53	16.84	8	0.27	33.3%	2.67
	Architecture	8	5.54	7.48	41	0.20	24.7%	2.68
	Lodging	1	15.53	16.84	8	0.37	46.8%	3.53
	Productivity	8	29.66	29.94	3	0.05	11.6%	3.53
	Productivity	8	29.94	31.32	5	0.06	14.0%	3.79
Goiás	Architecture	1	15.53	16.84	8	0.20	24.4%	2.66
	Architecture	8	5.54	7.48	41	0.19	23.2%	3.77
	Lodging	1	15.53	16.84	8	0.38	49.4%	3.33
	Productivity	1	38.68	40.50	31	0.12	40.0%	2.89
Paraná	Architecture	9	20.54	22.34	33	0.18	22.0%	2.58
	Architecture	11	48.52	50.00	24	0.22	26.8%	2.86
	Lodging	1	15.53	16.84	8	0.30	34.5%	2.69
	Productivity	4	24.31	25.25	4	0.10	17.5%	3.29

**^£^** percentage of *h*^2^ trait explained by RHM QTL (*h*^2^_RHM_/*h*^2^_G_); Chrom: chromosome. ^€^ Limits where the QTL genomic regions start and ends.

**Figure 3:**
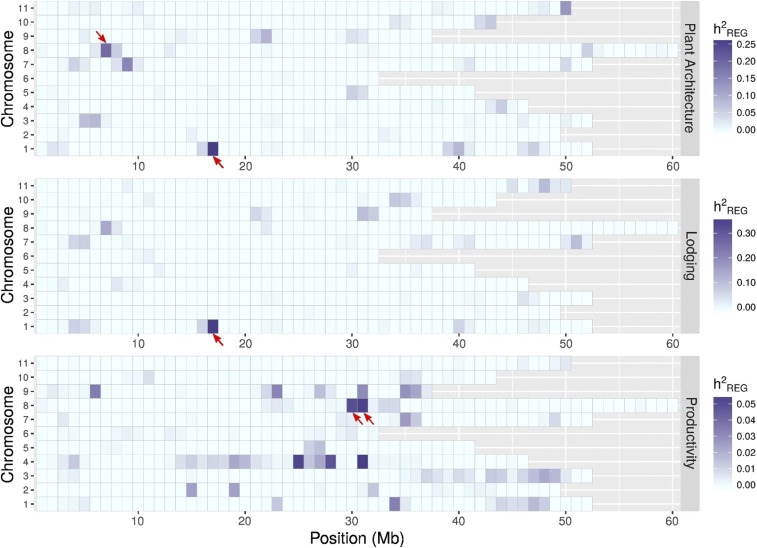
Genome-wide distribution of the significant QTL detected in the joint analysis by RHM along the 11 common bean chromosomes (y-axis) subdivided in 1 Mb windows for the three traits (right). Bar legends on the right correspond to regional heritability estimates. Red arrows indicate significant regions according to LRT test.

In Figure 3, a heat map shows RHM QTL distribution along the genome in both environments, with their respective heritability estimates presented. In addition to the RHM QTL that exceeded the significance threshold (red arrow), suggested additional associations along the genome are also represented in the heat map, such as the region labeled in dark blue on chromosome 4 above the interval of 25.25 Mb for the trait productivity.

### Comparative analysis Between the GWAS and RHM results

Whereas the GWAS detected eight significant markers, the RHM method detected 222 significant markers distributed among 13 genomic regions (Table 4). Although from the statistical point of view, all the QTL had a 90% confidence interval (α=0.05) for the threshold, in general, the values estimated for the GWAS QTL were considerably larger (ranging from 3.95 to 4.89) than those estimated for the RHM QTL (2.58 to 3.79). This is most evident when we examine the GWAS QTL identified at position 15.72 on chromosome 1 for architecture and lodging whose minimum threshold was 3.95 *vs.* 2.66 for the RHM QTL.

For the estimates of genomic heritability for both environments and the joint analysis, the GWAS-derived QTL explained approximately 23.2–28.4% of the genetic variance for architecture, 20.7–30.4% for lodging and 20.0–23.3% for productivity. In comparison, for the estimates obtained for the RHM-derived QTL, the levels of explained variance were higher, ranging from 47.6–57.6% for architecture, 34.5–46.8% for lodging and 14–40% for productivity.

**Figure 4:**
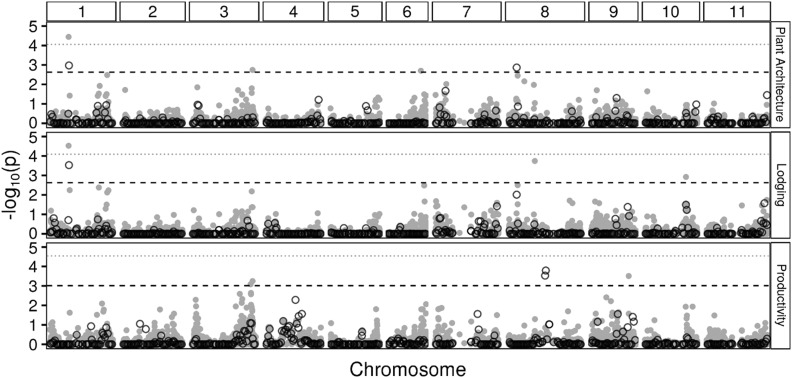
Manhattan plot of results from GWAS in gray solid circles and RHM in black unfilled circles for the tree traits (plant architecture, lodging and grain productivity). Lines indicate thresholds of genome-wide significance after permutation tests at 5% Bonferroni correction. Gray dotted, GWAS; black dashed, RHM.

Total genomic heritability using the full set of markers ($h_{SNP+DArT}^{2})$ captured between 72.09% and ≈100% of the heritability estimated from germplasm data ($h_{total}^{2}$). Larger fractions were captured for architecture and lodging when compared with productivity (72%). The fraction of genomic heritability explained by RHM analysis was higher when compared to GWAS for both traits, architecture (58.02% *vs.* 28.3%) and lodging (46.83% *vs.* 30.3%) (Table 4). Of the three different QTL identified by GWAS, one (position 15.72) was contained in an RHM region identified for the same traits (architecture and lodging). An integrated view of all QTL identified by GWAS and RHM in both environments is provided in a Manhattan plot (Figure 4). Several regions presented peaks at the same positions for both analyses but did not reach the significance threshold.

The average effect of allelic substitutions was evaluated considering the change in mean trait value when an allele was replaced at the QTL for lodging (DArT X3366188). We observed that allelic substitution in the QTL_GWAS_ significantly affected the lodging tolerance in both environments (S2 Fig).

### Gene identification in QTL regions

Annotation analysis for architecture- and lodging-associated markers (SNPs and DArTs) identified by joint analysis placed the QTL in chromosome 1 at position 15.53 to 16.84 with eight markers on a haplotype block spanning approximately 1.31 Mb. This block contained 40 transcripts, of which 32 were annotated in 24 genes and eight transcripts with no predicted function (S2 Table). For the QTL associated exclusively with architecture on chromosome 8 at position 5.54 to 7.48 and containing 41 markers, a total of 206 transcripts were identified, of which 185 were annotated in 167 putative genes. For the regions containing the QTL associated with productivity placed at positions 29.66 to 29.94 and 29.94 to 31.32, six and 24 transcripts were identified, annotated in 4 and 17 putative genes, respectively.

## Discussion

### Combined dominant and co-dominant markers for common bean

A predominance of homozygous loci is expected for the common bean, a preferentially autogamous species of which low outcrossing rates have been reported (Ferreira *et al.* 2007; Faria *et al.* 2010). In the present study, most estimates of heterozygosity were below 1% throughout the 11 chromosomes of common bean (Table 2), consistent with the self-fertilizing reproductive system of the species and in accordance with previously reported (1.3%) by others (Cardoso *et al.* 2014). Regarding the biological basis of the analyzed markers, *i*n this study DArT was scored as dominant (presence or absence of a probe target in the genome) and the conversion of these markers into a co-dominant pattern was possible due to the predominantly homozygous genome (alleles, AA and Aa, associated with presence and scored as dominant). The use of both types of markers produced by DArTseq provided complementary input data for our GWAS and RHM analysis, thus increasing the genome coverage and consequently the power of QTL detection (17,850 total markers evaluated). The large gaps (regions without marker coverage) identified along chromosomes in the present study were coincident with centromeric regions previously estimated for common bean (Schmutz *et al.* 2014). These genomic regions are expected to be gene-poor regions containing multiple and specific satellite repeats, with reduced recombination (Schmutz *et al.* 2014; Iwata *et al.* 2013), which makes them largely refractory based on the method of complexity reduction during DArT library preparation. Theoretically, the application of sequencing technology, which does not involve restriction enzymes, could considerably increase the representativeness of markers along the genome.

The genetic structure in Mesoamerican and Andean gene pools was confirmed based upon SNP and DArT data. In addition, the patterns of LD (*r*^2^) defined both by individual marker class and using the combined data set were very similar, as shown by the LD-decay curves (Table 2; Figure 2). Thus, the use of a less expensive and easier to obtain SNP marker wouldn´t have reduced these analyses’ power to detect true associations between markers and quantitative trait loci in beans. Of the 17,850 markers, 37.4% were in LE in this population, and a regional mapping window of 1 Mb was calibrated for the RHM analysis. For RHM the density of markers within a given window affects the structure of the regional-based genomic relationship matrix (GRM). Thus, would be expected that a window with more markers would also be more explanatory taking into account the possibility to observe high covariances in GRM. In this context, we opted to define the window length according to LD extent over SNP distances. Thus, it was possible to ensure that RHM was not directly affected by window length, since the “degree of information” provided by LD extent was equally distributed among the windows. In relation to GWAS, since the markers were individually tested over the genome, the marker effect significance was not directly affected by this density. However, under this last approach, care should be taken since the poor coverage of SNP panel implies in more noticeable lack of information because the absence of LD concept in the statistical analysis.

Knowledge of the genetic control of bean architecture and related traits are of great value in the development of effective strategies for selection. Architecture refers to the degree of branching, internode elongation, and shoot determinacy, among others (Wang and Li 2008). When the stem is insufficient to prevent displacement of the plant from the vertical axis, lodging occurs, causing major constraints to the production of several crops (Kashiwagi *et al.* 2008; Yamaguchi *et al.* 2014). In this study, the heritability estimates for plant architecture (81%) and lodging (79%) were considered of high magnitude and were consistent with results previously obtained for common bean for plant architecture (∼72.9%) (Alvares *et al.* 2016), and for lodging (78%) (Moghaddam *et al.* 2016). Therefore, in addition to the high potential of the population under study for selection of these traits with predominance of additive genetics effects, more progress will be made in selection due mostly to genes, which is adequate for the marker-assisted selection (MAS). For plant architecture and lodging, the combined set of markers explained most of the additive heritability for the traits (≈100%), indicating that, theoretically, most relevant markers were located in one, or few linked window, and the every segregant QTL was bracketed by markers.

For grain yield, the most important trait evaluated for common bean and other grain crops, a wide range of heritability values has been reported, which may be due to population effects, environmental factors or experimental accuracy. It has been estimated at ranges of 23–71% (Alvares *et al.* 2016; Gonçalves-Vidigal *et al.* 2008; Balcha 2014), in agreement with the result found in our study (43%). Moreover, the estimated genomic heritability *(h^2^_SNP+DArT_)* also captured a relatively large proportion (72%) of total trait heritability (*h*^2^_total_), which will be of great value in identifying favorable alleles for grain yield. Although the SNP analysis alone explained little more of the total genetic heritability (*h^2^_SNP_=*74%), the DArT and SNP combination (*h^2^_SNP+DArT_* = 72%) provided a double genome coverage, increasing the power to detect QTL by taking advantage of LD between causal variants and markers.

### Associations for plant architecture, lodging and grain productivity

In general, the proportion of genomic heritability explained by RHM for architecture, lodging and productivity was considerably higher (35.48 to 58.02) than that explained by GWAS (28.39 to 30.37) (Table 4). For plant architecture and lodging, which showed high trait heritability (81% and 79%, respectively), RHM identified more genome-wide significance accounting for larger fractions of the additive genetic variance regions than did GWAS (S3 Table). As proposed by others (Resende *et al.* 2017), this improvement resulted from the RHM analytical method, which combines interval mapping with association analysis to capture variance across the whole population, powered by the combined effect of several closely linked loci at the target locus. In RHM, while a significant single locus can be overshadowed by many non-significant loci within the same window, the combined effect of significant loci in high LD between them tend to highlight the window in question. In GWAS, a significant single locus tends to be highlighted independently of the LD pattern between it and the other loci. In addition, RHM avoids false positive associations identified by GWAS since a significant marker from GWAS does not implies in significant window under RHM framework (Riggio *et al.* 2013).

Based on GWAS, only one association was identified for the traits architecture and lodging, in both environment and joint analysis (chrom. 1 at 15.72 Mb). This was maintained for the lodging trait, with the same locus association observed for GWAS (explained 30%) and RHM (explained 46.8%) and hits on the same chromosome position with high consistency. As a significant correlation (0.7454) between lodging and architecture was identified, it reinforces that these mechanisms are associated and may be exploited together in further studies. Additional QTL for lodging were reported using the common bean biparental mapping population, of which four identified loci accounted for 39% of the genetic variance (Beattie *et al.* 2003) and two QTL accounted for 9% (Kolkman and Kelly 2003). More recently, based on GWAS analysis, a strong association for lodging was identified at chrom. 7 (46 Mb) that explained 21% of the phenotypic variation (Moghaddam *et al.* 2016). Compared with the high estimated values of lodging heritability (∼79%), we observed that not all loci acting to control this trait were identified in isolated studies, including ours. High heritability and low environmental effect, rather than providing information about the genetic architecture of the trait, it implies a greater correlation between phenotype and genotype (Visscher *et al.* 2008). The multiple different genomic regions associated with lodging suggest a trait with more genes involved.

Architecture is a complex trait related to several morphological traits with evidence of environmental influence and predominantly additive genetic control (Silva *et al.* 2013). In the 1990s, based on RAPD markers five QTL for plant uprightness and branch density were described (Jung *et al.* 1996). Posteriorly, using an increased set of markers (RAPD, SSR, STS, SCAR) six QTL for architecture-related traits (angle, height and hypocotyl diameter) explaining from 10.6 to 29.9 of the variance were reported (Beattie *et al.* 2003), followed by seven more QTL for general plant height and plant width, explaining from 8 to 19% (Blair *et al.* 2006). In such cases, QTL are difficult to compare, as they often employ different types of architecture measurements, but a consensus regarding a plant architecture QTL placed on chromosome 1 has been reported. In the present study, QTL for plant architecture was detect by both methods although RHM has captured twice as much heritability (58%). In addition, a QTL for architecture overlapped with a lodging QTL on chromosome 1, for which high heritability was found in the Goiás environment (38%) and in joint analysis (37%), being suggestive of a potential pleiotropic effect in such traits with benefits for breeding.

To date, several QTL for important productivity-related traits in common bean have been identified, including seed weight, pods per plant, seeds per plant, and days to flowering (Mukeshimana *et al.* 2014; Wright and Kelly 2011). More recently, QTL studies based on linkage and GWAS analysis using a larger set of SNPs have provided clearer insight into the genetic basis of agronomic traits related to productivity in common bean (Kamfwa *et al.* 2015; Hoyos-Villegas *et al.* 2016). In the present study, QTL associated with grain yield were identified in both GWAS and RHM analysis on chromosomes 1, 3, 4 and 8. Through GWAS, two QTL on chromosome 3 were identified in Goiás (explaining 30.23% of the variance), in accordance with previous GWAS (Mukeshimana *et al.* 2014) and linkage map analysis (Blair *et al.* 2006; Wright and Kelly 2011). Two RHM QTL on chromosome 8, identified in both environments in the present study, were in accordance with significant SNP associations previously identified by Kamfwa *et al.* (2015) based on GWAS, supporting the evidence for the presence of an important QTL conditioning seed yield at this position, or the presence of a substantial linkage disequilibrium among closely linked marker loci. In addition, environment-specific QTL on chromosomes 1 and 4 were reported in this study, in accordance with a number of QTL accounting for larger proportions of the phenotypic variance in seed yield previously identified through linkage analysis on chromosome 1 (Trapp *et al.* 2014) and 4 (Blair *et al.* 2006).

For all traits evaluated in the present study, high threshold values [–log_10_(p)] were reported for GWAS QTL, but these QTL explained lower amount of genetic variance than those identified by RHM. The differences of permutation-based significance threshold between RHM and GWAS are mainly due to LRT (Likelihood Ratio Test) values, while in GWAS the markers are fitted as fixed effects, in RHM are fitted as random effects with variance attributed to predefined windows containing a variable number of SNPs (Valdisser *et al.* 2017). Thus, the likelihood functions (constructed from these distinct models) are different; and consequently, the magnitude of LRT values is also naturally different; even under the randomization realized by the same permutation procedure **(**α=0.05*)*. However, the probabilistic significance level (reflected in the threshold value) is assured to be the same for both approaches. At present, both strategies of QTL identification lacked the power to detect all the QTL variance as estimated by trait heritability at the established threshold. Therefore, a considerable proportion of heritability, corresponding to the proportion of genetic variation that was not significantly detected and declared, is still missing. Figures 3 and 4 show several suggestive regions found with RHM that did not have large enough effects to be declared significant at the genome-wide level. The weak signals detected in the 100 kb SNP window form a basis for further analyses using smaller window sizes (Resende *et al.* 2017). The proportion of unexplained genetic variation could be attributed to imperfect LD between markers and QTL (de los Campos *et al.* 2015; Leamy *et al.* 2017), since the extend of LD is variable along the genome (Hyten *et al.* 2007). This suggests that the mapping resolution for RHM analysis could be improved using smaller windows (Nagamine *et al.* 2012). In addition, even with correction for population structure in the GWAS and RHM models to avoid unbiased results, the present study’s sample size may have contributed to reducing the power of analysis. A small sample size would have an even greater impact if causal allele variants are rare and provide a small average contribution to the phenotypic variance. The Mesoamerican and Andean gene pools had divergent processes of domestication and different geographic distributions accounting for a strong population structure and variable levels of genetic diversity (Bitocchi *et al.* 2012; Schmutz *et al.* 2014). Preferably, these populations should be evaluated separately, using a larger sample size to represent most of the available genetic variation and more representative genome-wide SNPs to increase the representation of all causal variants and provide more accurate estimates of LD. This is particularity important for traits with polygenic inheritance, for which this scenario would help to capture further associations with small effects underlying these traits and to find more precise estimates of QTL heritability (Uemoto *et al.* 2015).

### Genes in the QTL regions

Previous studies have reported a QTL for lodging on chromosome 1, as described through a linkage analysis combining three populations of common bean (R^2^ = 12.93) (Moghaddam *et al.* 2016). Additionally, a lodging QTL identified in the present study was consistently identified for the same trait on soybean, chromosome 19 (qLS19-1; R^2^ = 19.8%), which is syntenic with common bean chromosome 1 (Yamaguchi *et al.* 2014). The group of genes (24) within the lodging and architecture QTL included transcription factors, proline-rich proteins, lipolytic enzyme, and plant hormones, most of them implicated in diverse aspects of plant growth and development and mechanical resistance, providing support for the hypothesis that co-expression of these genes affects the lodging and architecture traits (Dharmasiri *et al.* 2005; Riemann *et al.* 2007). The SNP allelic variants associated with these traits are unlikely to be functional variants themselves because they were not placed within genes. Rather, based on LD, they could be used to determine the haplotype containing the functional variant, which would become the focus for follow-up studies. A molecular marker associated with lodging and architecture would be of great interest to select accessions from gene banks, to evaluate segregant populations and inbred line conversion in controlled (greenhouse) conditions where plants are inadequately characterized in lodging and architecture potential. In addition, the information of low correlation between plant architecture and productivity indicated that a marker will be useful on selections of plants carrying the favorable allele, and also to fix it in their homozygous state in early generations. Under this scenario, care should be taken, since the major gene is only one that affect the trait, and no marker selection has been applied to the other genes, which contributes to the overall phenotype (Muir and Stick 1998).

The positive QTL identified in the present study were derived from a genetically diverse group of germplasm, including many landraces that represent potentially useful materials for breeding efforts due to carrying different and potentially favorable alleles in architecture, lodging and yield-related traits. In addition, high-resolution chromosome analysis allowed increased precision in QTL mapping, because the associated variants are in LD with causal variants, opening new perspectives for MAS. By examining genes surrounding the QTL for productivity on chromosome 8, several candidate genes that may modulate plant growth and development, plant structure, cell cycle and morphogenesis were identified (Craig and Tyers 1999; Chepyshko *et al.* 2012). Adjacent QTL regions with several SNPs clustered and placed in an interval of 1.6 Mbp were detected by the RHM analysis, which represent an interesting candidate region for further investigation toward MAS.

### Conclusions

In this study, three traits were evaluated in two important common bean growing environments in Brazil, enabling the identification of QTL, which is the first step toward the development of a useful molecular resource for marker-aided selection (MAS) breeding. Particularly for lodging (total *h*2 ≥ 34.5 ≤ 49.4), the genome region identified (by GWAS and RHM) with environmental stability and a significant effect of allelic substitution represents the starting point for applying MAS aiming to increase the allele frequency at this locus in the breeding germplasm. On the other hand, the traits plant architecture and grain productivity, where a high number of QTL were identified (by RHM) and some were environment-specific, present a greater challenge to breeders. Genomic regions that did not reach the significance threshold to be declared QTL (Figures 3 and 4) were also suggestive of potential associations across the genome. Thus, by taking advantage of high resolution SNP genotyping and RHM’s effectiveness for identifying a large number of trait-associated markers, approaches that consider whole-genome effects and incorporate these data into predictive models, such as genome selection (GS), will exert great impact on breeding efficiency (Spindel *et al.* 2016; Zhang *et al.* 2015; Biazzi *et al.* 2017). As more genomic information becomes available, more data will be applied into common bean improvement, creating new opportunities for development and selection of elite breeding genotypes.
